# Xylitol-supplemented nutrition enhances bacterial killing and prolongs survival of rats in experimental pneumococcal sepsis

**DOI:** 10.1186/1471-2180-8-45

**Published:** 2008-03-11

**Authors:** Marjo Renko, Päivi Valkonen, Terhi Tapiainen, Tero Kontiokari, Pauli Mattila, Matti Knuuttila, Martti Svanberg, Maija Leinonen, Riitta Karttunen, Matti Uhari

**Affiliations:** 1 Department of Paediatrics, University of Oulu, Oulu, Finland; 2 Oral and Maxillofacial Department, Oulu University Hospital, Oulu, Finland; 3 Department of Dentistry, University of Oulu, Oulu, Finland; 4 National Public Health Institute, Oulu, Finland; 5 Department of Medical Microbiology, University of Oulu, Oulu, and Central Hospital of Kanta-Häme, Hämeenlinna, Finland

## Abstract

**Background:**

Xylitol has antiadhesive effects on *Streptococcus pneumoniae *and inhibits its growth, and has also been found to be effective in preventing acute otitis media and has been used in intensive care as a valuable source of energy.

**Results:**

We evaluated the oxidative burst of neutrophils in rats fed with and without xylitol. The mean increase in the percentage of activated neutrophils from the baseline was higher in the xylitol-exposed group than in the control group (58.1% vs 51.4%, P = 0.03 for the difference) and the mean induced increase in the median strength of the burst per neutrophil was similarly higher in the xylitol group (159.6 vs 140.3, P = 0.04). In two pneumococcal sepsis experiments rats were fed either a basal powder diet (control group) or the same diet supplemented with 10% or 20% xylitol and infected with an intraperitoneal inoculation of *S. pneumoniae *after two weeks. The mean survival time was 48 hours in the xylitol groups and 34 hours in the control groups (P < 0.001 in log rank test).

**Conclusion:**

Xylitol has beneficial effects on both the oxidative killing of bacteria in neutrophilic leucocytes and on the survival of rats with experimental pneumococcal sepsis.

## Background

Xylitol is a five-carbon polyol sugar widely distributed in plants, e.g. plums, strawberries and raspberries [1]. In humans, it is metabolised in the liver to glucose, glycogen and lactic acid. It affects blood glucose levels less than does glucose [2], and it has been used as a component in parenteral nutrition [3,4]. Xylitol has anticatabolic and antiketogenic effects and improves nitrogen balance in rats during septic infection [5,6]. This anabolic effect is thought to be the cause of the improved survival seen after thermal injury [7,8] and sepsis of intestinal origin [5].

Xylitol is an unsuitable source of energy for many micro-organisms, and it inhibits the growth of *Streptococcus pneumoniae *[9] and some other bacteria even in the presence of glucose, but not in the presence of fructose [10-12]. It has antiadhesive effects on both *S. pneumoniae *and *Haemophilus influenzae *[13] which are mediated by its effect on the cell wall of the bacteria [14]. Xylitol reduces the incidence of dental caries when given orally in chewing gum on a regular basis [15], and it has been found to be effective in preventing acute otitis media by up to 42% when administered either in chewing gum or as a syrup [16,17]. It causes a marked reduction in the intracellular redox state due to the rapid production of NADH and NADPH in the polyol dehydrogenase reaction [18], which may improve the oxidative burst and bacterial killing in polymorphonuclear leucocytes.

To explore the mechanisms of action of xylitol further, we measured the neutrophil respiratory burst related to bacterial oxidative killing in rats fed with or without xylitol. Having hypothesised that xylitol may be beneficial in modulating pneumococcal sepsis, we also evaluated the effect of xylitol-supplemented enteral nutrition on the course of pneumococcal sepsis in two rat experiments.

## Methods

### Animals and their nutrition

Six-week old male Sprague-Dawley rats were used throughout. They were fed a basal powder diet, RM1 (Special Diet Services, Witham, Essex, Great Britain), one kilogramme of which contains 885 g cereal products (wheat, barley and wheat feed), 60 g vegetable proteins, 25 g animal proteins (whey powder), 5 g soya oil, 7.1 g calcium, 2.9 g phosphorus, and 15 μg cholecalciferol. A list of the other minor components of the diet is given in the manufacturer's brochure. The rats in the xylitol groups were fed the same diet supplemented with 10% or 20% xylitol (w/w) (Xyrofin Co., Kotka, Finland) for two weeks before sampling in the burst trial and for two weeks before the bacterial challenge and during the induced disease in the survival experiments. The rats were kept in separate cages in a temperature and light-controlled room (21–23°C, 12-hour light-dark cycle) and had free access to tap water. The protocols were approved by the Ethical Committee on Animal Experiments of the University of Oulu.

### Respiratory burst test

The respiratory burst that reflects bacterial oxidative killing was measured using a commercial test (Bursttest, Orpegen Pharma, Heidelberg, Germany) according to the manufacturer's instructions. The test is based on the principle that reactive oxygen mediates the transformation of dihydrorhodamine 123 to rhodamine 123, which can be detected by flow cytometry. Both the percentage of neutrophils with respiratory burst activity and the average strength of the activity per neutrophil were analysed. The respiratory burst was induced by *E. coli *in a standardized manner. The baseline activity was measured in a tube containing only buffer without bacteria and subtracted from the induced response in each sample. A human blood sample was tested with the rat samples in each series to confirm the performance of the test. The results were analyzed by FACScan flow cytometry (Becton-Dickinson) using the FACScan program.

**Figure 1:**
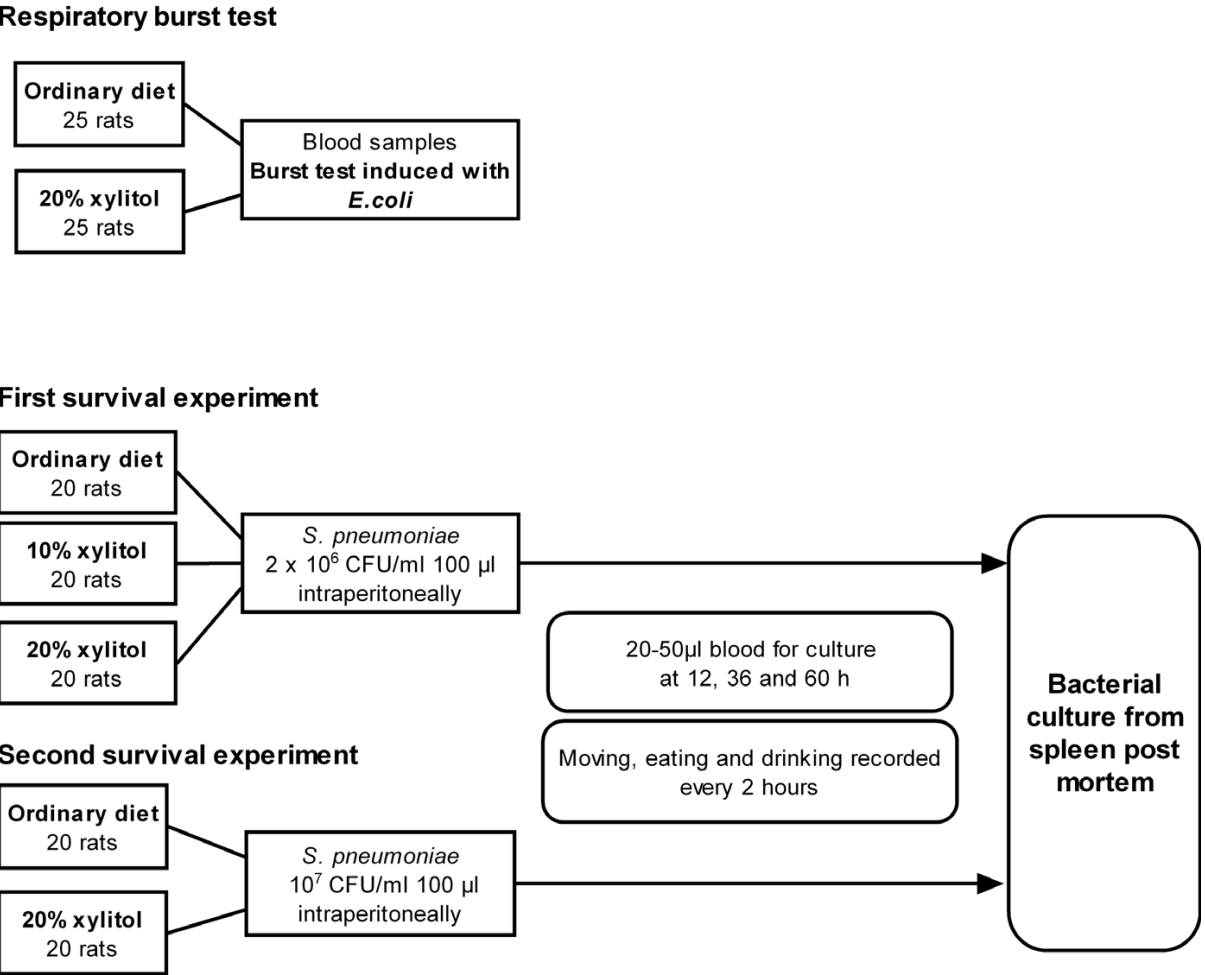
The protocol of the burst test and first and second survival experiments, showing the diet groups, inoculation protocol and follow-up of the animals.

Fifty animals were assigned to two groups of 25 to receive either a control diet or the same diet supplemented with 10% xylitol for one week and 20% xylitol for the second week (Figure 1). After two weeks a blood sample of 5 ml was taken from the heart under Hypnorm-Dormicum anaesthesia just before the rats were killed with an overdose of carbon dioxide and severing of the head. The respiratory burst test was performed as described above.

### Survival experiments

In the first survival experiment sixty rats were assigned to three groups of 20 each, receiving either a basal powder diet or the same diet supplemented with 10% or 20% xylitol (Figure 1). The mean weight of the animals was 171 g (range 127 – 266) before the diet and 214 g (157 – 280) at the time of the inoculation. The mean weight gain resulting from the experimental diet was greater in the control animals (55.7 g, p < 0.01) than in those receiving 10% xylitol (30.4 g) or 20% xylitol (35.4 g), but the mean weights of the animals before inoculation did not differ significantly between the groups.

In the second experiment forty rats were assigned to two groups of 20, receiving either a control diet or the same diet supplemented with 20% xylitol (Figure 1).

### Bacterial strains and culture conditions

The rats were infected with *S. pneumoniae *type 3 (patient isolate, National Public Health Institute strain collection, Oulu, Finland), which had been stored at -80°C in skimmed milk. After overnight incubation on a blood agar plate in an atmosphere containing 5% CO_2 _at +37°C, several colonies were inoculated into Brain Heart Infusion medium (Difco Laboratories, Detroit, MI, USA) and again incubated overnight at +37°C. The bacteria were then collected by centrifugation for 15 min at 1500 × g and the precipitate suspended in phosphate buffer, pH 7.2, until the desired density was obtained with the McFarland density standards. The final bacterial concentration was confirmed by a standard plate dilution method on a blood agar plate.

### Bacterial challenge and samples

After two weeks on the experimental diet, the animals were infected by intraperitoneal inoculation with 100 μl of the bacterial suspension containing 2 × 10^6 ^colony-forming units (CFU)/ml (first experiment) or 2 × 10^7 ^CFU/ml (second experiment). Blood samples were taken by suborbital puncture 12, 36 and 60 hours post-inoculation. A sample of 50 μl was diluted immediately with 50 μl of sterile 0.9% NaCl and spread on a gentamycin blood agar plate. The number of pneumococcal colonies was counted after overnight incubation at + 37°C and classified as 1) negative, 2) positive with less than 10^2 ^CFU/ml, 3) positive with 10^2 ^to 10^3 ^CFU/ml, or 4) positive with more than 10^3 ^CFU/ml.

Serum concentrations of xylitol were measured in blood samples taken 12 hours after the challenge. The samples were kept at room temperature for 5 hours and centrifuged at 1500 × g for 15 min, after which the serum was collected. The concentration of xylitol in each sample was measured by enzymatic assay using the polyol dehydrogenase-based procedure of Boehringer Mannheim (Mannheim, Germany). Because of the small amounts of serum available, some measurements were performed by pooling 4–5 samples within each diet group (Table 1). The detection limit of the assay was 0.07 mmol/L (10 μg/mL).

**Table 1:** Serum samples, numbers of animals in pools and xylitol concentrations in 20 rats fed a diet with 20% xylitol supplementation.

Sample	Total no. of animals	Xylitol concentration (mmol/L)
1 – 4	10	< 0.07
5	1	0.07
6	3	0.07
7	6	0.13

The animals were monitored visually at intervals of about 2–4 hours and the experimental diet was continued throughout the trial. The spleens of the deceased rats were cultured to ensure that death had been caused by pneumococcal sepsis. Pneumococci in the blood and spleen cultures were identified by their typical colony morphology and optochin sensitivity.

### Statistical analyses

The sample size for the burst test trial was estimated by assuming that the mean difference in the percentage of neutrophils in which the respiratory burst occur between the xylitol and control group would be 2.5%. With a power of 80% and an alpha error of 0.05 a sample size of 21 rats per group was needed. Since some of the tests might fail, it was decided that the final size of both groups should be 25 rats. Mean differences with 95% confidence intervals were calculated for continuous variables in the xylitol and control groups and the statistical significances were tested with Students' t-test. The significance in the differences of proportions between the groups was tested with the SND (standard normal deviation) test. The mean and median survival times with 95% confidence intervals (CI) were calculated, and the survival times of the groups were calculated by the Kaplan-Meier method, the differences being tested with the Log Rank test.

## Results

### Respiratory burst

**Figure 2:**
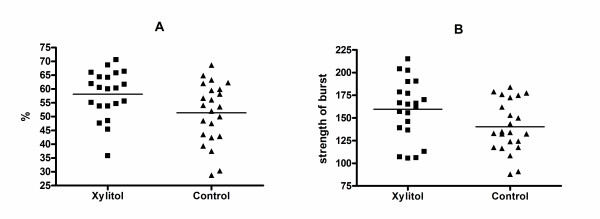
**Increase in the percentage of activated neutrophils from the baseline (A) and increase in the median value for the strength of the burst (B) when induced with *E. coli *in rats fed with and without xylitol**. The lines indicate mean values.

The mean increase from the baseline in the percentage of activated neutrophils was 58.1% in the xylitol group (n = 21) and 51.4% in the control group (n = 23) (mean difference 6.7%, CI 0.7–12.8, P = 0.03 for the difference) (Figure 2A). Correspondingly, the mean induced increase in the median value of the strength of the burst at the cell level was 159.6 in the xylitol group and 140.3 in the control group (mean difference 19.3, CI 0.7–37.9, P = 0.04 for the difference) (Figure 2B). The results of six samples were not available due to missed incubation time.

### Survival experiments

#### Blood and spleen cultures

All the animals with positive blood cultures that died during the follow-up also had highly positive spleen cultures for pneumococci post mortem. Six animals that remained apparently healthy during the follow-up were killed 109 hours after the challenge. Blood and spleen cultures were negative except that for a few pneumococcal colonies growing in the blood culture of one animal and one colony in the spleen culture of another, both in the xylitol group. When the results of both experiments were combined, the blood cultures 12 hours after inoculation were significantly more often negative in the 20% xylitol groups than in the control group (17.5% vs 2.5%, P = 0.03).

All the blood and spleen cultures from the animals that died from obvious clinical sepsis showed the growth of abundant, slimy colonies typical of type 3 *S. pneumoniae*. Five randomly chosen colonies were serotyped and identified as type 3. By contrast, the colonies isolated from the blood or spleen of the animals in the xylitol group without clinical signs of sepsis were small and dry and could not be serotyped.

#### Survival time

**Figure 3:**
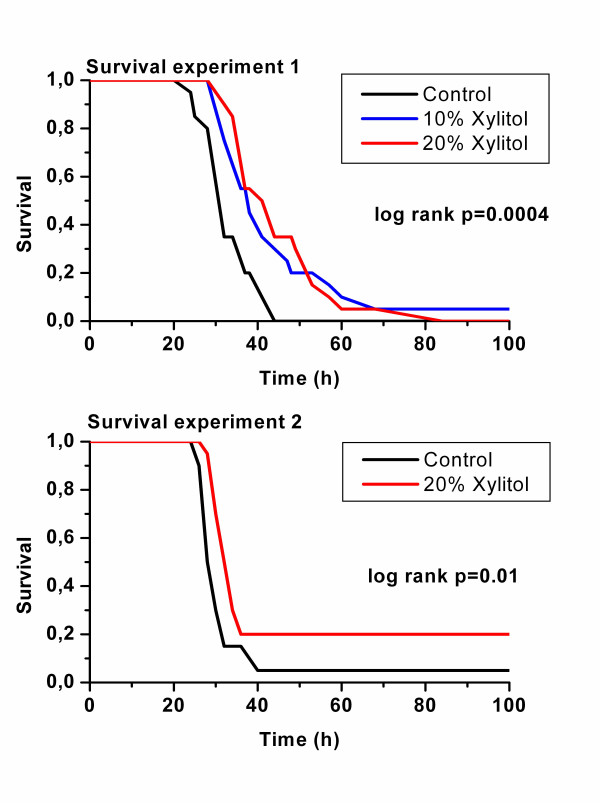
Survival of the animals after intraperitoneal inoculation with *S. pneumoniae*, by diet group in experiments 1 and 2.

In both experiments the animals with sepsis in the xylitol groups survived longer than those in the control groups, the mean survival times being 34 hours (CI 30 to 38, median 32 hours) in the control groups and 46 hours (40 to 51, median 36 hours) in the xylitol groups. In experiment 1 the mean survival time was 33 hours in the control group (CI 31 to 36, median 32 hours), 44 hours in the 10% Xylitol group (CI 37 to 51, median 38) and 45 hours in the 20% Xylitol group (CI 40 to 50, median 41, P value in log rank test 0.0004, Figure 3). In experiment 2 the mean survival time was 34 hours in the control group (CI 26 to 41, median 28 hours) and 47 hours in the 20% Xylitol group (CI 34 to 61, median 32, P value in log rank test 0.01.

#### Concentration of xylitol in serum

No detectable amount of xylitol was found in the serum of any of the animals in the control group or the 10% xylitol group, whereas a concentration of 1.3 mmol/L was present in one pooled sample from the 20% xylitol group and a level just above the detection limit was seen in two other samples (Table 1).

## Discussion

We were able to show that dietary xylitol enhanced oxidative killing in neutrophilic leucocytes and prolonged the survival of rats suffering from sepsis caused by *S. pneumoniae *type 3. These findings are in accordance with a previous report which showed that parenteral xylitol improved the survival of rats suffering from intestinal sepsis [5]. This better survival in the presence of sepsis probably entails several contributory factors. Xylitol has effects on the growth [9], expression of capsular polysaccharides [14] and adhesion [13] of *S. pneumoniae*. It has also been shown to have several beneficial metabolic effects in animals suffering from trauma or sepsis [5-8], and the enhanced functioning of neutrophilic leucocytes found in the present study provides an extension to this list.

The initiation of the respiratory burst in polymorphonuclear neutrophils requires NAPDH. Xylitol may affect the metabolism of neutrophils in two ways: firstly by improving the glycaemic control under the hyperglycaemic stress reaction during sepsis, or secondly by causing a marked reduction in the intracellular redox state due to the rapid production of NADH and NADPH in the polyol dehydrogenase reaction [18]. By thus affecting the energy metabolism of the leukocytes, it may interfere with their functioning, and thus modulate the course of the invasive bacterial disease. Xylitol has also been shown to be cytoprotective during oxidative stress [19].

*S. pneumoniae *is one of the most common bacteria causing invasive infections in otherwise healthy persons. The maximal expression of capsular polysaccharides is known to be essential for its systemic virulence, however, because of the antiphagocytic properties of the capsule [20]. We have shown earlier that the ultrastructure of the pneumococcal capsule becomes ragged after exposure to xylitol [14]. Interestingly, two pneumococcal isolates from xylitol-fed rats with no clinical signs of septicaemia in the present study grew rough colonies and were impossible to serotype, suggesting that the virulent type 3 pneumococci had become avirulent, not expressing the genes needed for capsular synthesis. The *regM *gene, encoding catabolite control protein, has recently been identified in pneumococcus [21]. This gene is involved in the catabolism of carbohydrates, and its mutation resulted in a significant reduction in the transcription of the locus of capsular polysaccharide biosynthesis. Further studies would be needed, however, to show, whether a xylitol diet can lead to repression of the regM gene and consequently to reduced capsular synthesis.

The serum concentrations of xylitol recorded here were low, as was to be expected, because xylitol is absorbed slowly from the gut by free diffusion, the rate of absorption being 1/5 of that of glucose [22]. Xylitol is able to inhibit the growth of pneumococci significantly at a concentration of 65 mmol/L (1%) [9], and concentrations of the same magnitude are expected for the morphological changes [14]. The highest serum concentration recorded in this experiment was 1.3 mmol/L, in the 20% xylitol group, we did not measure intra-tissue or intracellular concentrations, nor do we know whether or not the serum concentrations reflect the amount of xylitol present during the critical steps in the infection process.

Ardawi^5 ^found that xylitol enhances survival in sepsis of intestinal origin, where the growth or virulence of the aetiological microbes is not known to be susceptible to xylitol. Parenteral xylitol has been shown to have a nitrogen-sparing effect during sepsis and after burns in rats [5,6], and Ardawi [5] suggested that this may be the mechanism for improved survival in the presence of sepsis. The nitrogen balance may nevertheless be only an indirect sign of a better state, reflecting less serious inflammation, and not the reason for the improvement in survival.

Oral xylitol is well tolerated in adults and children, the only side-effect found being osmotic diarrhoea [22]. Parenteral xylitol can cause minimal hyperuricaemia, but without any pathophysiological consequences [3]. Xylitol has been used as a component of parenteral nutrition, instead of or in addition to glucose, e.g. in patients with diabetes [3,4]. Though tolerated well in modest doses, large doses of xylitol administered intravenously have been reported to cause renocerebral oxalosis with renal failure [23,24]. These case reports of serious side-effects raise a need for caution in the use of parenteral xylitol.

The factors influencing the severity of invasive bacterial infections are quite well known, but only a few methods of treatment aimed at slowing them down are in clinical use. Xylitol, as a modulator of the functioning of leukocytes during the inflammatory process, may open up new possibilities for improving the prognosis for pneumococcal sepsis.

## Conclusion

Xylitol has beneficial effects on both the oxidative killing of bacteria in neutrophilic leucocytes and on the survival of rats with experimental pneumococcal sepsis.

## Authors' contributions

MR did the statistics and wrote the first form of the manuscript. MR and TK made the bacterial cultures in the survival studies and PV, TT and RK performed the burst tests. PM and MS took care of the animals. MK, ML and MU designed the study. All authors read and approved the final manuscript.
